# Supplementary material for: Myeloperoxidase Inhibition Ameliorates Plaque Psoriasis in Mice
Source: Antioxidants (Basel). 2021 Aug 25;10(9):1338. doi: 10.3390/antiox10091338 (PMC8472607; doi:10.3390/antiox10091338)
Supplement: Supplementary file 1 [file antioxidants-10-01338-s001.zip › antioxidants-1301464-supplementary.pdf]

Supplemental Figure S1

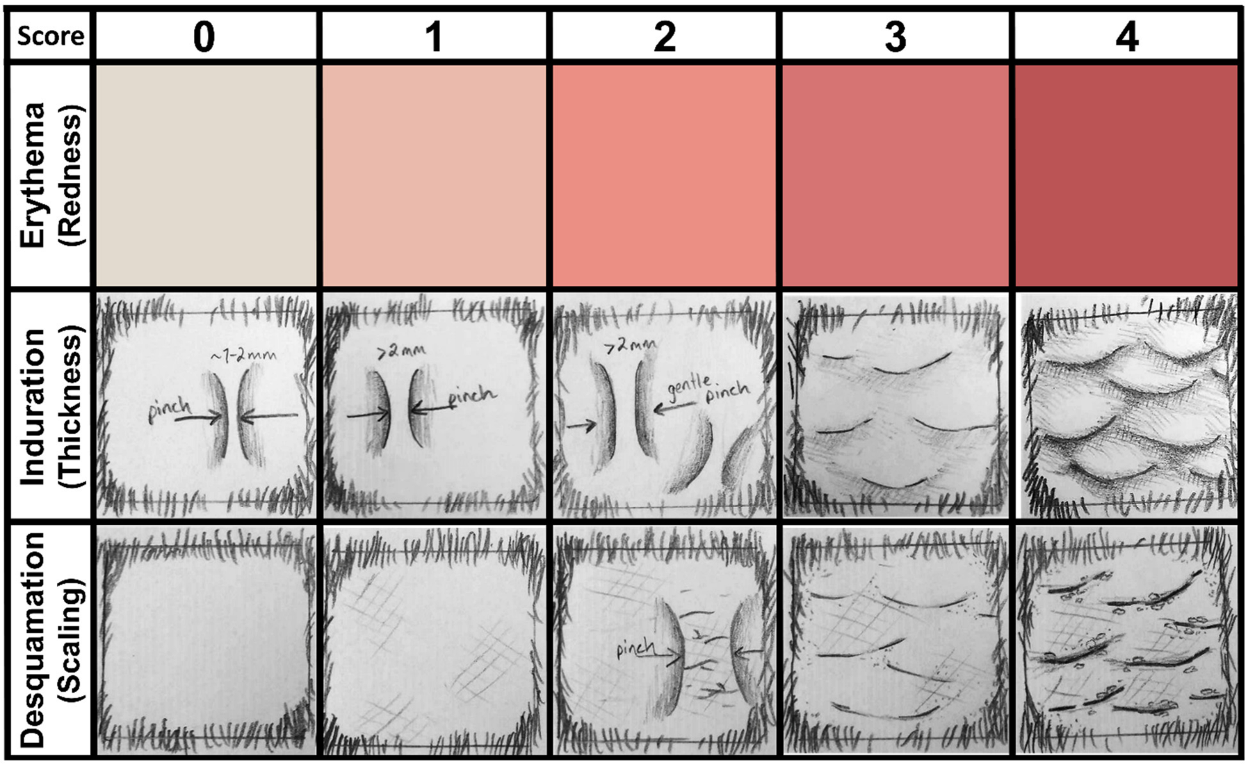

Figure S1. Drawn mouse PASI scoring system. Erythema, induration, and desquamation severity indices were drawn based on visual cues observed from mouse experiments to further describe the mouse PASI scale shown in Figure 2. This may be used alongside Table 1 in assessing skin inflammation on the mouse back skin.
